# Impact of sarcopenia on intertrochanteric femoral fracture in the elderly

**DOI:** 10.7717/peerj.13445

**Published:** 2022-06-15

**Authors:** Shunli Jiang, Yu Ding, Lixing Kang

**Affiliations:** 1 The Affiliated Lianyungang Oriental Hospital, Kangda College of Nanjng Medical University, Lianyungang, Jiangsu Province, China; 2 The Affiliated Lianyungang Oriental Hospital, Xuzhou Medical University, Lianyungang, Jiangsu Province, China; 3 Wafangdian Central Hospital, Dalian, Liaoning province, China; 4 Department of Orthopedics, Langfang People’s Hospital, Langfang, Hebei Province, China

**Keywords:** Femoral intertrochanteric fracture, Sarcopenia, Instrumental activities of daily living

## Abstract

**Objective:**

The aim of this study was to investigate the effect of skeletal sarcopenia on the prognosis of intertrochanteric fracture in the elderly.

**Methods:**

We collected information on 144 patients with femoral intertrochanteric fracture (FIF). The influence of sarcopenia on the chance of death was determined using binary Probit regression analysis. For additional analysis, the Chow test was utilized to select the best distinguishing node in the instrumental activities of daily living (IADL) score. We looked for characteristics that were linked to a higher probability of death and a poor IADL outcome within 1 year. The data collected above were analyzed using logistic regression analysis. The internal calibration degree and model validity were assessed by GiViTI calibration.

**Results:**

Sarcopenia, EuroQol-5D 1 month score, age, gender, and hypertension were identified as risk factors for death in older patients with FIF within a year by logistic regression analysis. Sarcopenia, psychotropics, BMI, and length of hospital stay were all found to be risk factors for poor IADL outcomes (*P* < 0.1). The calibration curves indicated that the anticipated and actual probabilities of these two models were very close. The study’s reliability coefficient was 0.671, showing a satisfactory level of reliability.

**Conclusion:**

In elderly patients with FIF, sarcopenia, EuroQol-5D score, age, gender, and hypertension were risk factors for death; sarcopenia, hospital stay length, BMI were risk factors for poor quality of life.

## Introduction

Intertrochanteric fracture is a fracture of the femoral neck from the base of the femur to just above the level of the lesser trochanter (Uzun, 2009; Butler et al., 2009). It is most common in the elderly. Fractures of the femoral trochanter often result in prolonged bed rest, which can lead to complications such as pneumonia and muscle atrophy, increasing the risk of death and reducing the quality of life in the elderly. Femoral intertrochanteric fracture (FIF) are usually treated surgically using rigid internal fixation (Uzun, 2009) and thereby avoiding a series of problems caused by prolonged bed rest (Weller et al., 2005). However, even with surgical treatment, the risk of postoperative death in these elderly patients is higher than in other elderly people. The prognosis of FIF elderly patients is affected by several factors (Peeters et al., 2016; Loggers et al., 2020).

Clinical observations have shown that elderly patients with FIF combined with sarcopenia often have a poor prognosis. The concept of sarcopenia was first introduced by Rosenberg in 1989 (Rosenberg, 1997). In 2010, the European Working Group on Sarcopenia in Older People (EWGSOP) defined sarcopenia as an age-related progressive reduction in whole-body skeletal muscle mass, strength, or physical function (Cruz-Jentoft et al., 2010). The examination of muscle mass is usually performed by bioelectrical impedance analysis (BIA), dual-energy x-ray absorptiometry (DXA), peripheral quantitative computed tomography (CT), and magnetic resonance imaging (MRI). Among them, BIA is widely used in clinical practice because of its low cost, safety, and convenience.

The incidence of sarcopenia increases with age (Cruz-Jentoft et al., 2019). Sarcopenia can seriously affect mobility in older adults. The prognosis for elderly patients with FIF in combination with sarcopenia is often poor (Landi et al., 2017; Yoo et al., 2018). However, there is no satisfactory way to predict mortality and quality of life after the surgery for femoral trochanteric fracture. The aim of this study was to investigate the effect of skeletal sarcopenia on the prognosis of intertrochanteric fracture in the elderly. Using a combination of clinical observations and literature review, we conducted a study suggesting that sarcopenia and several other factors affect mortality and quality of life in patients with FIF 1 year after surgery.

## Materials and Methods

### Population

We collected information on 144 patients with intertrochanteric fracture of the femur who attended the Department of Orthopedics, Langfang People’s Hospital from June 2016 to June 2020 in this cross-sectional study. We obtained patient data through the electronic medical records of patients aged >65 years and patients without cognitive impairment or language impairment and other conditions that might make it impossible for them to understand the questionnaire and complete follow-up information. The exclusion criteria were as follows: patients with major diseases or decompensation of vital organs, patients with bilateral fractures and other surgeries on the same side or malignancies and life-threatening conditions that occurred during the follow-up period, and patient data that the investigator determined were not credible. All included patients underwent standard treatments. All participants were informed of the study orally and in writing, and written informed consent was also obtained. The ethical procedures of the study procedures were in accordance with the Declaration of Helsinki. The study was supported by Langfang People’s Hospital Ethics Committee (IRB approval number 2016003). The clinical characteristics of each patient were collected, including age, sex, marital status, education, survival, body mass index, residential status, income, depression, sensory impairment, diabetes, hypertension, American Society of Anesthesiologists EuroQol-5D questionnaire score, presence of osteoporosis, instrumental activities of daily living score, and history of anti-osteoporosis medication use. Surveys were completed at admission and follow up (1, 6, and 12 months). All survey data were entered by two researchers and checked for accuracy.

### Diagnosis of sarcopenia

Bioelectrical impedance analysis (BIA) is an effective method for diagnosing sarcopenia (Wang et al., 2016; Chen et al., 2020). The appendicular skeletal muscle mass (ASM) of the extremities was measured by the BIA method using a body composition analyzer (TANITA-MC180) immediately after the patients were admitted with fractures, and muscle strength was assessed by ASM/H2. To complete the BIA measurement, the subjects held the electrode in their hand, with the body was resting on the hospital bed, and the foot was placed on the foot electrode. With the assistance of medical personnel, the patient completed the test in under a minute. This study adopted the Asian Working Group on Sarcopenia risk screening method and EWGSOP2 to define sarcopenia (Chen et al., 2014; Cruz-Jentoft et al., 2019). Therefore, muscle mass ≤7.0 kg/m^2^ in men and ≤6.0 kg/m^2^ in women was used as a diagnostic criterion for sarcopenia.

### EuroQol-5D questionnaire

The EuroQol-5D is a questionnaire that is widely used as a clinical and an economic assessment tool for the health-related quality of life (EuroQol Group, 1990). Each dimension of the EuroQol-5D scoring system is divided into three levels. Instrumental activities of daily living scores are completed using the Lawton and Brody scale (Lawton & Brody, 1969). These eight scoring items followed previous studies. Previous researched included these items included in the scale assessed were as previous research (Alekna et al., 2018).

### Statistical analysis

Statistical analysis was performed using SPSS version 22 software (IBM Corp., Armonk, NY, USA) and R software (version 3.5.3; R Core Team, 2019). The measurement data were expressed as mean plus/minus standard deviation and statistically analysed by an independent sample t-test; count data were expressed as percentages (%) and statistically analysed by the χ2 test; the non-parametric rank sum test was used for the grade information. Reliability analysis was used to study the reliability of the collected data (Eisinga, Grotenhuis & Pelzer, 2013). A Cronbach’s α coefficient higher than 0.6 indicates acceptable reliability and <0.6 indicates poor reliability. To analyze the effect of sarcopenia on the risk of death in older patients with FIF, we performed a binary Probit regression analysis using sarcopenia as the independent variable and death as the dependent variable. As described in previous studies, the Chow test was used to determine the best distinguishing node of the IADL score, by SAS9.3 software, allowing it to be further analyzed (Liang et al., 2017). A 10-fold cross-validation method using recursive feature elimination (RFE) with random forest as classifier and R-packet insertion was used to screen features associated with risk of death and adverse IADL outcomes within 1 year in patients with age-related FIF (Wang et al., 2020). Eventually, 144 samples were included in the analysis, and logistic regression analysis was performed with the characteristics obtained above as in previous studies (Kang et al., 2020; Zhou et al., 2021; Ying et al., 2021). To assess the internal calibration degree and the validity of the model, the givitiR package of the R software was used to plot the GiViTI calibration bands of this logistic model (Gebremariam et al., 2021). In this study, *P* < 0.05 was considered significant.

## Results

### Demographic characteristics of patients

Among the 144 participants, 35 patients (24.3%) had comorbid sarcopenia on admission, and there were 36 males and 108 females. The demographics of the participants are shown in Table 1. Body mass index (BMI), death, income, availability of chaperones, history of hip fracture, EuroQol-5D 1-month score, EuroQol-5D 6-month score, EuroQol-5D 12-month score, IADL 1-month score, IADL 6-month score, and IADL-12 month score were important risk factors for sarcopenia. The reliability coefficient value of the study is 0.671, which is greater than 0.6, indicating the acceptable quality of the study data for reliability (Table S1). Table 2 shows the binary Probit regression analysis that was performed with sarcopenia as the independent variable and death as the dependent variable, using the model equation Probit (P) = −2.210 + 0.822 × Sarcopenia (where p represents the risk of death within 1 year in patients with FIF). The results suggest that sarcopenia is a risk factor for poor prognosis in older patients with FIF. The marginal effect value was 0.162, which suggests that sarcopenia increases the mortality risk by 16.19% in older patients with FIF in short-term follow up.

**Table 1:** Demographic characteristics of the patients.

Items	Sarcopenia (*n* = 144)		Total (*n* = 144)	*P*-value	
	NO (*n* = 109)	YES (*n* = 35)			
Age		84.40 ± 6.38	86.29 ± 6.96	84.86 ± 6.55	0.14
BMI		26.24 ± 3.98	24.34 ± 3.00	25.78 ± 3.85	0.011*
Death	No	100 (91.74)	25 (71.43)	125 (86.81)	0.002**
	Yes	9 (8.26)	10 (28.57)	19 (13.19)	
Intervention	No	54 (49.54)	23 (65.71)	77 (53.47)	0.095
	Yes	55 (50.46)	12 (34.29)	67 (46.53)	
Gender	Female	82 (75.23)	26 (74.29)	108 (75.00)	0.911
	Man	27 (24.77)	9 (25.71)	36 (25.00)	
Illiteracy	No	74 (67.89)	21 (60.00)	95 (65.97)	0.391
	Yes	35 (32.11)	14 (40.00)	49 (34.03)	
Marital status	Married	35 (32.11)	11 (31.43)	46 (31.94)	0.94
	Single	74 (67.89)	24 (68.57)	98 (68.06)	
Availability of chaperones	Yes	19 (17.43)	12 (34.29)	31 (21.53)	0.035*
	No	90 (82.57)	23 (65.71)	113 (78.47)	
Income (yuan)	<500	1 (0.92)	4 (11.43)	5 (3.47)	0.020*
	500–1,000	92 (84.40)	24 (68.57)	116 (80.56)	
	1,000–1,500	13 (11.93)	6 (17.14)	19 (13.19)	
	>=1,500	3 (2.75)	1 (2.86)	4 (2.78)	
Intervention complications	No	92 (84.40)	30 (85.71)	122 (84.72)	0.851
	Yes	17 (15.60)	5 (14.29)	22 (15.28)	
Hypertension	No	25 (22.94)	9 (25.71)	34 (23.61)	0.736
	Yes	84 (77.06)	26 (74.29)	110 (76.39)	
Diabetes	No	71 (65.14)	27 (77.14)	98 (68.06)	0.185
	Yes	38 (34.86)	8 (22.86)	46 (31.94)	
Dyslipidemia	No	73 (66.97)	26 (74.29)	99 (68.75)	0.417
	Yes	36 (33.03)	9 (25.71)	45 (31.25)	
Osteoporosis	No	101 (92.66)	32 (91.43)	133 (92.36)	0.811
	Yes	8 (7.34)	3 (8.57)	11 (7.64)	
Previous hip fracture history	No	106 (97.25)	29 (82.86)	135 (93.75)	0.004**
	Yes	3 (2.75)	6 (17.14)	9 (6.25)	
Psychotropics	No	58 (53.21)	20 (57.14)	78 (54.17)	0.685
	Yes	51 (46.79)	15 (42.86)	66 (45.83)	
Bisphosphontes	No	106 (97.25)	35 (100.00)	141 (97.92)	0.321
	Yes	3 (2.75)	0 (0.00)	3 (2.08)	
Sensory disturbances	No	52 (47.71)	16 (45.71)	68 (47.22)	0.837
	Yes	57 (52.29)	19 (54.29)	76 (52.78)	
ASA score	I	1 (0.92)	0 (0.00)	1 (0.69)	0.945
	II	35 (32.11)	11 (31.43)	46 (31.94)	
	III	65 (59.63)	21 (60.00)	86 (59.72)	
	IV	8 (7.34)	3 (8.57)	11 (7.64)	
Depressive mood	No	49 (44.95)	14 (40.00)	63 (43.75)	0.607
	Yes	60 (55.05)	21 (60.00)	81 (56.25)	
EuroQol-5D 1-month score		53.30 ± 17.24	43.20 ± 16.64	51.28 ± 17.53	0.009**
EuroQol-5D 6-month score		59.20 ± 19.32	49.60 ± 22.36	57.28 ± 20.24	0.033*
EuroQol-5D 12-month score		60.20 ± 19.38	46.00 ± 19.84	57.36 ± 20.22	0.001**
IADL 1-month score		2.25 ± 1.40	1.36 ± 0.81	2.07 ± 1.35	*P* < 0.001**
IADL 6-month score		2.21 ± 1.74	1.64 ± 1.44	2.1 ± 1.7	0.134
IADL 12-month score		2.29 ± 1.88	1.32 ± 0.85	2.1 ± 1.77	*P* < 0.001**

**Notes:**

Measures are expressed as mean ± standard deviation; counts are expressed as data ± percentages.

ASA, American Society of Anesthesiologists; BMI, Body Mass Index; IADL, Instrumental activities of daily living.

* *P* < 0.05.

** *P* < 0.01.

**Table 2:** Summary of the results of the binary Probit regression analysis related to Death.

Items	Regression coefficient	standard error	Z value	*P* value	95% CI	Marginal effects
Sarcopenia	0.822	0.284	2.898	0.004	[0.266–1.378]	0.162
Intercept	−2.21	0.413	−5.354	*P* < 0.001	[−3.019 to −1.401]	–

**Notes:**

Dependent variable: Death.

McFadden R: 0.074.

Cox & Snell R: 0.056.

Nagelkerke R: 0.104.

### Determination of the optimal IADL node and screening of key prognostic features

**Figure 1:**
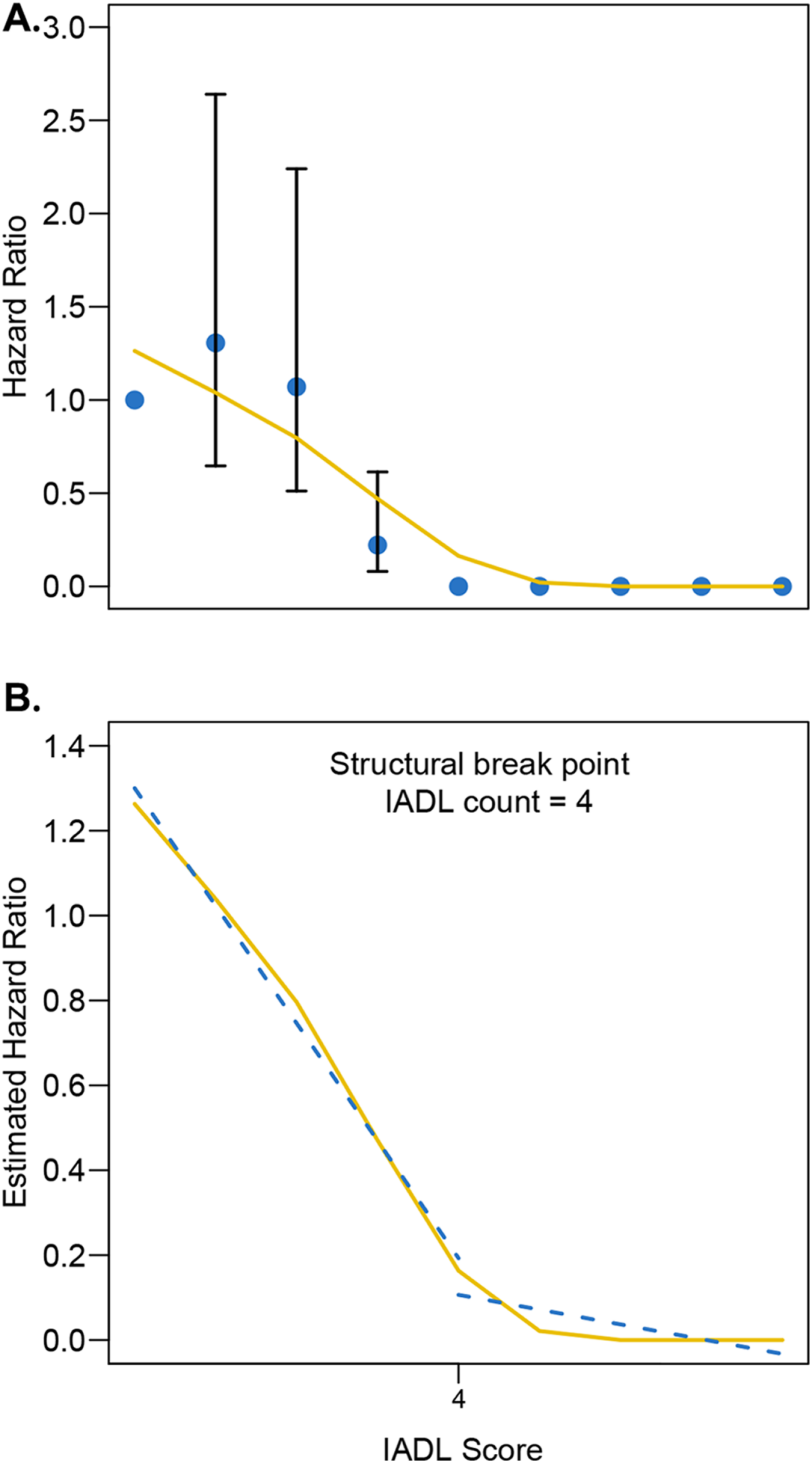
(A–B) The Chow test was used to establish the best differentiating node for the IADL score after accounting for sex, age, sarcopenia, and intervention mode. The Chow test was used to establish the best differentiating node for the IADL score after accounting for sex, age, sarcopenia, and intervention mode.

**Figure 2:**
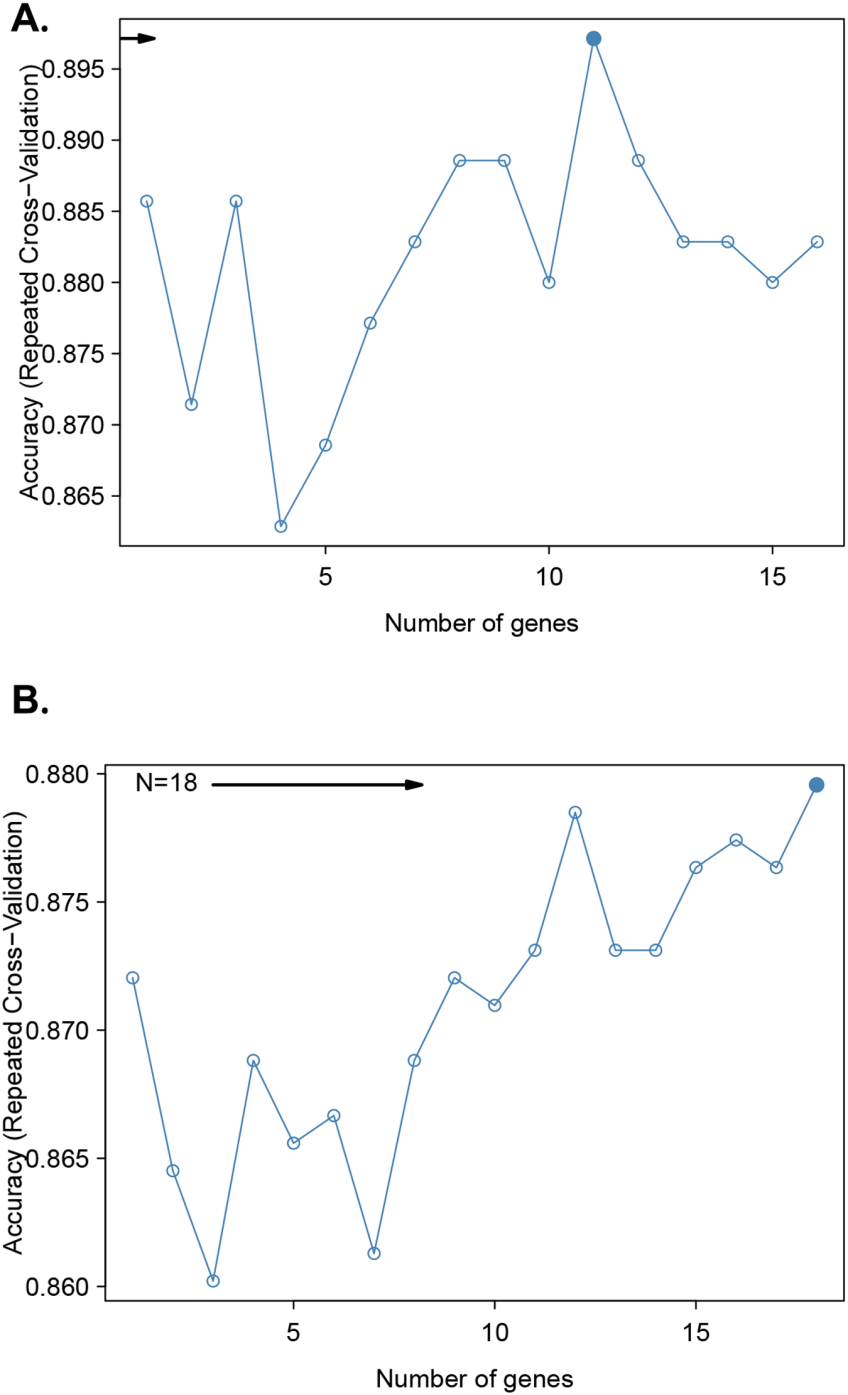
A recursive feature elimination algorithm was used to investigate the relationship between predictive accuracy and the number of selected features. Predictive accuracy for mortality risk within 1 year in older femoral intertrochanteric fracture patients (A) and predictive accuracy for poor IADL outcome (B).

After adjusting for sex, age, sarcopenia, and intervention modality, the Chow test was used to determine the best distinguishing node for the IADL score after femoral trochanter fracture in patients (Fig. 1A). An IADL score of four was considered to give the best discrimination. A recursive feature elimination algorithm based on the random forest model was used to analyze the relationship between the predictive accuracy and the number of selected features (Fig. 1B). The highest accuracy in predicting the mortality risk within 1 year in older patients with FIF was achieved by including 11 factors: EuroQol-5D 1-month score, EuroQol-5D 6-month score, EuroQol-5D 12-month score, age, sex, hypertension, psychotropics, intervention, marital status, sarcopenia, and depressive mood history (Fig. 2A). The highest accuracy in predicting IADL outcome in older patients with FIF was achieved by including 18 factors (Fig. 2B). The excessive number of parameters makes clinical work difficult, so we have selected the 11 most relevant ones here to improve clinical usability (including hospital stay duration, sarcopenia, age, illiteracy, depressive mood, BMI, dyslipidemia, psychotropics, diabetes, availability of chaperones, and intervention).

### Muscular sarcopenia is associated with mortality and reduced quality of life within 1 year after FIF in older patients

**Figure 3:**
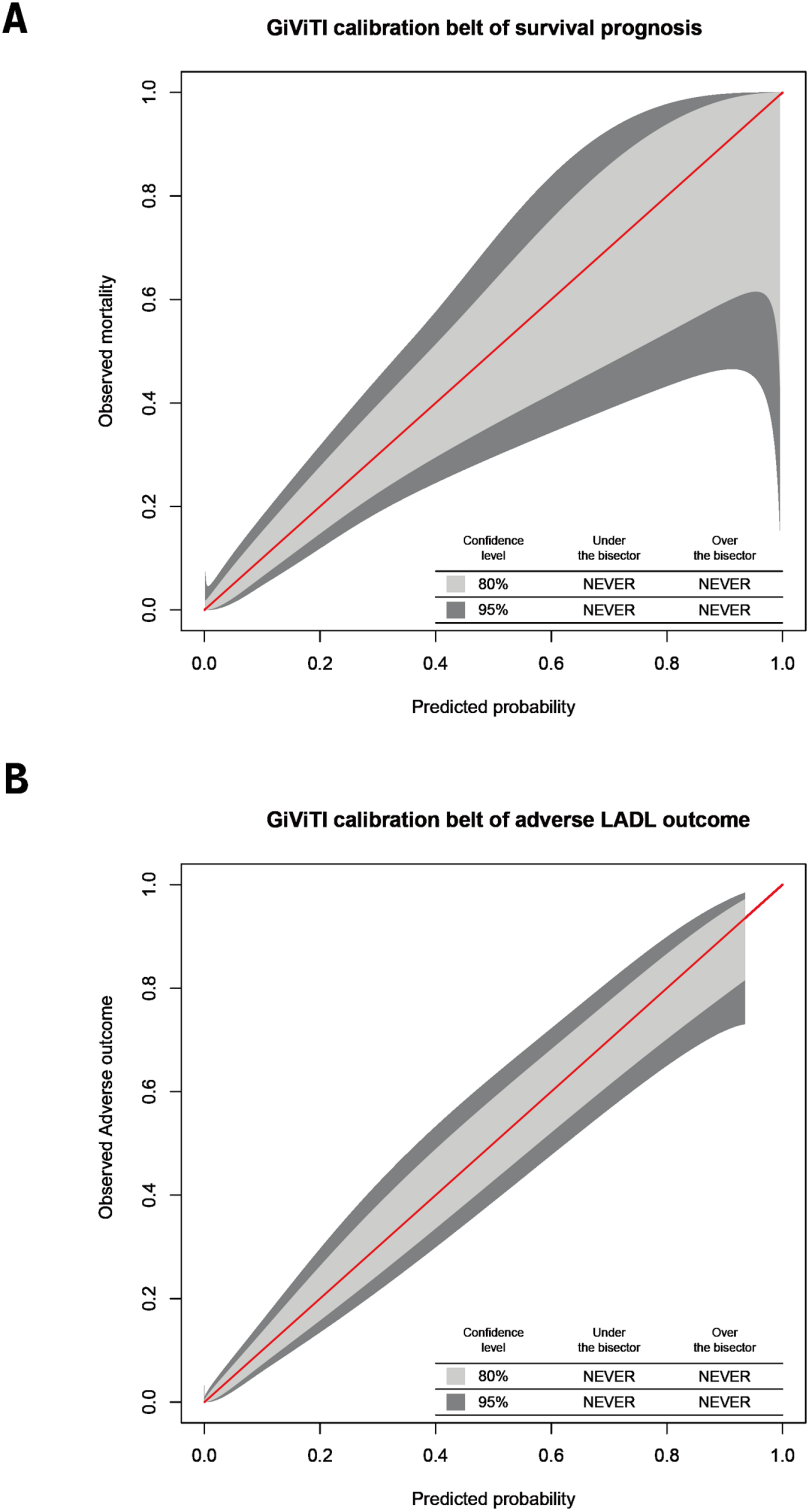
Calibration plots. In older individuals with femoral intertrochanteric fracture, calibration plots of logistic regression models for 1-year mortality risk (A) and unfavorable IADL outcome (B).

The variables screened from the random forest model were included in the unconditional binary logistic regression analysis, which showed that the EuroQol-5D 1-month score, age, sex, hypertension, and sarcopenia were risk factors for death within 1 year in elderly patients with FIF (*P* < 0.1; Table 3). Hospital stay duration, sarcopenia, BMI, and psychotropics were risk factors for adverse IADL outcomes in patients with FIF (*P* < 0.1; Table 4). The calibration curves of the logistic regression models for the risk of death within 1 year and adverse IADL outcome in older patients with FIF suggested a strong agreement between the predicted and actual probabilities of these two models (Figs. 3A and 3B). In conclusion, sarcopenia is a common risk factor for adverse IADL outcomes and death within 1 year in older patients with FIF.

**Table 3:** Prediction factors for mortality risk.

Variable	Prediction model		
	β	Odds ratio (95% CI)	*P*-value
(Intercept)	−2.279	0.102 [0–95.853]	0.514
EuroQol-5D 1-month score	0.058	1.06 [1.017–1.104]	0.006
EuroQol-5D 6-month score	0.046	1.047 [0.977–1.121]	0.191
EuroQol-5D 12-month score	0.038	1.039 [0.971–1.111]	0.267
Age	−0.089	0.915 [0.842–0.993]	0.033
Gender	−1.475	0.229 [0.076–0.692]	0.009
Hypertension	−1.056	0.348 [0.134–0.902]	0.030
Psychotropics	−0.367	0.693 [0.279–1.721]	0.429
Intervention	0.251	1.285 [0.535–3.085]	0.574
Marital status	−0.574	0.563 [0.205–1.549]	0.266
Sarcopenia	−1.990	0.137 [0.015–1.225]	0.075
Depressive mood history	0.334	1.396 [0.49–3.981]	0.533

**Note:**

β is the regression coefficient.

**Table 4:** Prediction factors for adverse LADL outcome.

Variable	Prediction model			
	β	Odds ratio (95% CI)		*P*-value
(Intercept)	−7.200	0 [0–6.87]		0.122
Hospital stay time	0.290	1.34 [1.08–1.67]		0.008
Sarcopenia	1.450	4.25 [1.22–14.77]		0.023
Age	0.010	1.01 [0.92–1.1]		0.861
Illiteracy	0.580	1.79 [0.54–5.99]		0.344
Depressive mood	−0.460	0.63 [0.2–1.99]		0.434
BMI	0.140	1.15 [0.98–1.35]		0.088
Dyslipidemia	−1.000	0.37 [0.08–1.76]		0.211
Psychotropics	−1.270	0.28 [0.07–1.07]		0.063
Diabetes	−0.910	0.4 [0.09–1.85]		0.243
Availability of chaperones	−1.120	0.33 [0.08–1.28]		0.108
Intervention	−0.870	0.42 [0.12–1.54]		0.190

**Note:**

BMI, Body Mass Index; β is the regression coefficient.

## Discussion

This study confirms that sarcopenia is a common risk factor for death and decreased quality of life among elderly patients with FIF and is the greatest risk factor for increased mortality and decreased quality of life.

Sarcopenia was diagnosed by BIA, and the quality of life of patients was quantified using EuroQol-5D and IADL scores. A binary Probit regression analysis with sarcopenia as the independent variable and death as the dependent variable yielded the model equation Probit (P) = −2.210 + 0.822* sarcopenia (where p represents the risk of death within 1 year in patients with FIF). This condition indicated that sarcopenia is a risk factor for death within 1 year among elderly patients with FIF and increases the risk by 16.19%. The unconditional binary logistic regression analysis revealed that the five risk factors (*P* < 0.1) for death within 1 year among elderly patients with FIF were EuroQol-5D 1 month score, age, sex, hypertension and sarcopenia, and the four risk factors (*P* < 0.1) for adverse IADL outcome among elderly patients with FIF were the length of hospital stay, sarcopenia, BMI and psychotropics. Therefore, sarcopenia is a common risk factor for death and decreased quality of life postoperatively within 1 year among elderly patients with FIF.

EuroQol-5D is a critical metric for determining a patient’s quality of life. In this study, patients without concomitant sarcopenia after intertrochanteric fracture had a EuroQol-5D 1 month score of 0.53, a EuroQol-5D 6 month score of 0.59, and a EuroQol-5D 12 month score of 0.60. Patients’ EuroQol-5D scores were greater than in a previous fracture injury study (Vu et al., 2019a). This could be a result of China’s rapid development in medical care in recent years, which, along with effective preoperative and postoperative care, has resulted in patients having better overall EuroQol-5D scores. Patients without concomitant sarcopenia had EuroQol-5D scores that were similar to fragile elderly patients in a previous study (EuroQol-5D = 0.58) (Vu et al., 2019b). Patients with sarcopenia had a EuroQol-5D 1-month score of 0.43, a EuroQol-5D 6-month score of 0.49, and a 12-month EuroQol-5D score of 0.46, which was similar to the earlier study of the elderly after a fall injury (Vu et al., 2019b). Sarcopenia appears to be a risk factor for femur intertrochanteric fractures. Furthermore, EuroQol-5D was lower in patients after a greater intertrochanteric fracture of the femur than after dengue fever (EuroQol-5D = 0.66), respiratory diseases (EuroQol-5D = 0.66), skin diseases (EuroQol-5D = 0.73), human immunodeficiency virus (HIV) (EuroQol-5D = 0.8), diabetes (EuroQol-5D = 0.8), and the general population under COVID-19 (EuroQol-5D = 0.95) (Tran et al., 2018b, 2018a, 2020; Nguyen et al., 2018, 2019). We found that the EuroQol-5D 1-month score was a significant risk factor for 1-year mortality.

The Kaplan–Meier estimate found that sarcopenia did not affect the 1-year mortality (*P* = 0.793) but significantly affected the 5-year mortality (*P* = 0.028) (Kim et al., 2018). Yoo et al. (2018) compared the effects of both sarcopenia and osteoporosis among elderly patients with hip fracture, accounting for 7.8% in general, 10.3% in combination with sarcopenia, 15.1% in combination with both sarcopenia and osteoporosis, and 5.1% in patients with osteoporosis only (Yoo et al., 2018). Unlike other studies that included osteoporosis as an influencing factor for mortality, our study revealed that osteoporosis alone did not increase mortality among elderly patients with hip fracture, and mortality was significantly higher in patients with concomitant sarcopenia and osteoporosis. Previous studies have also concluded that sarcopenia is not associated with postoperative mortality among elderly patients with FIF (Di Monaco et al., 2015; González-Montalvo et al., 2016; Chang et al., 2018).

Unlike previous studies, our study compared the effects of sarcopenia on the 1-year mortality among elderly patients with FIF in China, showing that 28.57% of patients with skeletal sarcopenia and osteoporosis died within 1 years, which was much higher than the 8.26% in those with FIF alone. Not only was sarcopenia confirmed as a significant risk factor for increased 1-year mortality and reduced quality of life among elderly patients with FIF, but also four other risk factors for higher 1-year mortality (EuroQol-5D 1-month score, age, sex and hypertension) and three risk factors for reduced quality of life (length of hospital stay, BMI and use of psychotropics) were identified. This finding provides information on additional interventions to reduce mortality and improve the quality of life of elderly patients with FIF.

Femoral intertrochanter fractures are known as the last fracture in life and are of concern because of their high post-fracture mortality and serious impact on quality of life. This study further confirms that sarcopenia is an important risk factor for mortality or reduced quality of life after a FIF among elderly patients, and sarcopenia can be prevented or improved by increasing the nutritional intake and exercise training; therefore, the following factors should be considered: (1) pay attention to the muscle quality of the elderly individuals to prevent sarcopenia and (2) screen elderly patients with sarcopenia among those with FIF to detect and intervene in sarcopenia promptly to reduce improve quality of life and mortality among elderly patients with FIF. Generally, the muscle mass is assessed with dual-energy X-ray imaging, BIA, CT or MRI (Heymsfield et al., 2015). Among them, CT or MRI measures the cross-sectional area of the skeletal muscle of the body to calculate the skeletal muscle index (SMI) for a more accurate diagnosis of sarcopenia (Nemec et al., 2017). However, CT exposes the patients to a higher radiation dose, and MRI is expensive and takes a long time to carry out; therefore, we used BIA to diagnose sarcopenia, which may overestimate the muscle mass because of metal implants in the lower extremity among patients with FIF and may impact the accuracy of the test results. Furthermore, this research was not a prospective randomized controlled clinical trial, and the pre-fracture quality-of-life scores of elderly patients with FIF could be determined only after a hospital admission, which inevitably resulted in recall bias. Comparing these scores (EuroQol-5D and IADL) with values from other diseases/studies is also a worthwhile study to be conducted in the future.

## Conclusion

Sarcopenia is a risk factor for death and decreased quality of life in elderly patients with FIF within 1 year after surgery, which opens up new avenues for lowering the risk of death and addressing the problem of decreased quality of life in elderly patients with FIF after surgery.

## Supplemental Information

10.7717/peerj.13445/supp-1 Supplemental Information 1 Cronbach’s Reliability Analysis.

10.7717/peerj.13445/supp-2 Supplemental Information 2 Raw data.

## Additional Information and Declarations

### Data Availability

The following information was supplied regarding data availability: The raw measurements are available in the Supplemental Files.

## References

[ref-1] Alekna V, Kilaite J, Mastaviciute A, Tamulaitiene M (2018). Vitamin D level and activities of daily living in octogenarians: cross-sectional study. Frontiers in Endocrinology.

[ref-2] Butler M, Forte M, Kane RL, Joglekar S, Duval SJ, Swiontkowski M, Wilt T (2009). Treatment of common hip fractures. Evidence Report/Technology Assessment.

[ref-3] Chang C-D, Wu JS, Mhuircheartaigh JN, Hochman MG, Rodriguez EK, Appleton PT, Mcmahon CJ (2018). Effect of sarcopenia on clinical and surgical outcome in elderly patients with proximal femur fractures. Skeletal Radiology.

[ref-4] Chen Y, Cai Y, Kang X, Zhou Z, Qi X, Ying C, Zhang Y, Tao J (2020). Predicting the risk of sarcopenia in elderly patients with patellar fracture: development and assessment of a new predictive nomogram. PeerJ.

[ref-5] Chen L-K, Liu L-K, Woo J, Assantachai P, Auyeung T-W, Bahyah KS, Chou M-Y, Chen L-Y, Hsu P-S, Krairit O, Lee JSW, Lee W-J, Lee Y, Liang C-K, Limpawattana P, Lin C-S, Peng L-N, Satake S, Suzuki T, Won CW, Wu C-H, Wu S-N, Zhang T, Zeng P, Akishita M, Arai H (2014). Sarcopenia in Asia: consensus report of the Asian Working Group for Sarcopenia. Journal of the American Medical Directors Association.

[ref-6] Cruz-Jentoft AJ, Baeyens JP, Bauer JM, Boirie Y, Cederholm T, Landi F, Martin FC, Michel J-P, Rolland Y, Schneider SM, Topinková E, Vandewoude M, Zamboni M, European Working Group on Sarcopenia in Older People (2010). Sarcopenia: European consensus on definition and diagnosis: report of the European working group on sarcopenia in older people. Age and Ageing.

[ref-7] Cruz-Jentoft AJ, Bahat G, Bauer J, Boirie Y, Bruyère O, Cederholm T, Cooper C, Landi F, Rolland Y, Sayer AA, Schneider SM, Sieber CC, Topinkova E, Vandewoude M, Visser M, Zamboni M (2019). Writing Group for the European Working Group on Sarcopenia in Older People 2 (EWGSOP2), and the Extended Group for EWGSOP2, Sarcopenia: revised European consensus on definition and diagnosis. Age and Ageing.

[ref-8] Di Monaco M, Castiglioni C, De Toma E, Gardin L, Giordano S, Di Monaco R, Tappero R (2015). Presarcopenia and sarcopenia in hip-fracture women: prevalence and association with ability to function in activities of daily living. Aging Clinical and Experimental Research.

[ref-9] Eisinga R, Grotenhuis M, Pelzer B (2013). The reliability of a two-item scale: pearson, cronbach, or spearman-brown?. International Journal of Public Health.

[ref-10] EuroQol Group (1990). EuroQol-a new facility for the measurement of health-related quality of life. Health Policy.

[ref-11] Gebremariam AD, Tiruneh SA, Engidaw MT, Tesfa D, Azanaw MM, Yitbarek GY, Asmare G (2021). Development and validation of a clinical prognostic risk score to predict early neonatal mortality, Ethiopia: a receiver operating characteristic curve analysis. Clinical Epidemiology.

[ref-12] González-Montalvo JI, Alarcón T, Gotor P, Queipo R, Velasco R, Hoyos R, Pardo A, Otero A (2016). Prevalence of sarcopenia in acute hip fracture patients and its influence on short-term clinical outcome. Geriatrics & Gerontology International.

[ref-13] Heymsfield SB, Gonzalez MC, Lu J, Jia G, Zheng J (2015). Skeletal muscle mass and quality: evolution of modern measurement concepts in the context of sarcopenia. The Proceedings of the Nutrition Society.

[ref-14] Kang X, Chen B, Chen Y, Yi B, Yan X, Jiang C, Wang S, Lu L, Shi R (2020). A prediction modeling based on SNOT-22 score for endoscopic nasal septoplasty: a retrospective study. PeerJ.

[ref-15] Kim YK, Yi SR, Lee YH, Kwon J, Jang SI, Park SH (2018). Effect of sarcopenia on postoperative mortality in osteoporotic hip fracture patients. Journal of Bone Metabolism.

[ref-16] Landi F, Calvani R, Ortolani E, Salini S, Martone AM, Santoro L, Santoliquido A, Sisto A, Picca A, Marzetti E (2017). The association between sarcopenia and functional outcomes among older patients with hip fracture undergoing in-hospital rehabilitation. Osteoporosis International.

[ref-17] Lawton MP, Brody EM (1969). Assessment of older people: self-maintaining and instrumental activities of daily living. The Gerontologist.

[ref-18] Liang W, He J, Shen Y, Shen J, He Q, Zhang J, Jiang G, Wang Q, Liu L, Gao S, Liu D, Wang Z, Zhu Z, Ng CSH, Liu C, Horsleben Petersen R, Rocco G, D’Amico T, Brunelli A, Chen H, Zhi X, Liu B, Yang Y, Chen W, Zhou Q, He J (2017). Impact of examined lymph node count on precise staging and long-term survival of resected non–small-cell lung cancer: a population study of the us seer database and a chinese multi-institutional registry. Journal of Clinical Oncology.

[ref-19] Loggers SAI, Van Lieshout EMM, Joosse P, Verhofstad MHJ, Willems HC (2020). Prognosis of nonoperative treatment in elderly patients with a hip fracture: a systematic review and meta-analysis. Injury.

[ref-20] Nemec U, Heidinger B, Sokas C, Chu L, Eisenberg RL (2017). Diagnosing sarcopenia on thoracic computed tomography: quantitative assessment of skeletal muscle mass in patients undergoing transcatheter aortic valve replacement. Academic Radiology.

[ref-21] Nguyen HTT, Moir M, Nguyen TX, Vu AP, Luong LH, Nguyen TN, Nguyen LH, Tran BX, Tran TT, Latkin CA, Zhang MW, Ho RC, Vu HTT (2018). Health-related quality of life in elderly diabetic outpatients in Vietnam. Patient Preference and Adherence.

[ref-22] Nguyen SH, Nguyen LH, Vu GT, Nguyen CT, Le THT, Tran BX, Latkin CA, Ho CSH, Ho RCM (2019). Health-related quality of life impairment among patients with different skin diseases in Vietnam: a cross-sectional study. International Journal of Environmental Research and Public Health.

[ref-23] Peeters CMM, Visser E, Van de Ree CLP, Gosens T, Den Oudsten BL, De Vries J (2016). Quality of life after hip fracture in the elderly: a systematic literature review. Injury.

[ref-37] R Core Team (2019). A language and environment for statistical computing. https://www.r-project.org.

[ref-24] Rosenberg IH (1997). Sarcopenia: origins and clinical relevance. The Journal of Nutrition.

[ref-25] Tran B, Dang A, Truong N, Ha G, Nguyen H, Do H, Nguyen T, Latkin C, Ho C, Ho R (2018a). Depression and quality of life among patients living with HIV/AIDS in the era of universal treatment access in Vietnam. International Journal of Environmental Research and Public Health.

[ref-26] Tran BX, Nguyen HT, Le HT, Latkin CA, Pham HQ, Vu LG, Le XTT, Nguyen TT, Pham QT, Ta NTK, Nguyen QT, Ho CSH, Ho RCM (2020). Impact of COVID-19 on economic well-being and quality of life of the vietnamese during the national social distancing. Frontiers in Psychology.

[ref-27] Tran B, Thu Vu G, Hoang Nguyen L, Tuan Le Nguyen A, Thanh Tran T, Thanh Nguyen B, Phuong Thi Thai T, Latkin C, Ho C, Ho R (2018b). Cost-of-Illness and the health-related quality of life of patients in the dengue fever outbreak in Hanoi in 2017. International Journal of Environmental Research and Public Health.

[ref-28] Uzun M (2009). Long-term radiographic complications following treatment of unstable intertrochanteric femoral fractures with the proximal femoral nail and effects on functional results. Acta Orthopaedica et Traumatologica Turcica.

[ref-29] Vu HM, Dang AK, Tran TT, Vu GT, Truong NT, Nguyen CT, Doan AV, Pham KTH, Tran TH, Tran BX, Latkin CA, Ho CSH, Ho RCM (2019a). Health-related quality of life profiles among patients with different road traffic injuries in an urban setting of Vietnam. International Journal of Environmental Research and Public Health.

[ref-30] Vu HM, Nguyen LH, Tran TH, Pham KTH, Phan HT, Nguyen HN, Tran BX, Latkin CA, Ho CSH, Ho RCM (2019b). Effects of chronic comorbidities on the health-related quality of life among older patients after falls in vietnamese hospitals. International Journal of Environmental Research and Public Health.

[ref-31] Wang H, Hai S, Cao L, Zhou J, Liu P, Dong B-R (2016). Estimation of prevalence of sarcopenia by using a new bioelectrical impedance analysis in Chinese community-dwelling elderly people. BMC Geriatrics.

[ref-32] Wang Q, Li M, Yang M, Yang Y, Song F, Zhang W, Li X, Chen K (2020). Analysis of immune-related signatures of lung adenocarcinoma identified two distinct subtypes: implications for immune checkpoint blockade therapy. Sedentary Life and Nutrition.

[ref-33] Weller I, Wai EK, Jaglal S, Kreder HJ (2005). The effect of hospital type and surgical delay on mortality after surgery for hip fracture. The Journal of Bone and Joint Surgery. British Volume.

[ref-34] Ying C, Guo C, Wang Z, Chen Y, Sun J, Qi X, Chen Y, Tao J (2021). A prediction modeling based on the hospital for special surgery (HSS) knee score for poor postoperative functional prognosis of elderly patients with patellar fractures. BioMed Research International.

[ref-35] Yoo JI, Kim H, Ha YC, Kwon HB, Koo KH (2018). Osteosarcopenia in patients with hip fracture is related with high mortality. Journal of Korean Medical Science.

[ref-36] Zhou S-P, Fei S-D, Han H-H, Li J-J, Yang S, Zhao C-Y (2021). A prediction model for cognitive impairment risk in colorectal cancer after chemotherapy treatment. BioMed Research International.

