# Supplementary material for: Impact of sarcopenia on intertrochanteric femoral fracture in the elderly
Source: PeerJ. 2022 Jun 15;10:e13445. doi: 10.7717/peerj.13445 (PMC9206433; doi:10.7717/peerj.13445)
Supplement: Supplemental Information 1 [file peerj-10-13445-s001.docx]

| Supplementary Table 1. Cronbach's Reliability Analysis | | | |
| --- | --- | --- | --- |
| Items | Correction Item Total Correlation (CITC) | α coefficient | Cronbach α coefficient |
| Sarcopenia | -0.257 | 0.673 | 0.671 |
| Death | 0 | 0.672 |  |
| Intervention | 0.171 | 0.671 |  |
| Age | -0.378 | 0.705 |  |
| Gender | -0.018 | 0.672 |  |
| Marriage status | -0.071 | 0.672 |  |
| Illiteracy | -0.118 | 0.672 |  |
| Coexistence | -0.385 | 0.677 |  |
| Income | -0.053 | 0.672 |  |
| Length of hospital stay | 0.071 | 0.671 |  |
| Intervention complications | -0.162 | 0.672 |  |
| Hypertension | -0.039 | 0.672 |  |
| Diabetes | -0.008 | 0.672 |  |
| Dyslipidemia | 0.085 | 0.671 |  |
| Osteoporosis | -0.012 | 0.672 |  |
| Previous hip fracture history | -0.006 | 0.672 |  |
| Psychotropics | -0.16 | 0.673 |  |
| Bisphosphonates | 0.025 | 0.672 |  |
| Sensory disturbances | -0.198 | 0.673 |  |
| ASA | -0.116 | 0.673 |  |
| Depressive | -0.205 | 0.673 |  |
| BMI | 0.1 | 0.67 |  |
| EuroQol-5D 1 month score | 0.793 | 0.559 |  |
| EuroQol-5D 6 month score | 0.908 | 0.524 |  |
| EuroQol-5D 12 month score | 0.9 | 0.527 |  |
| IADL 1 month score | 0.647 | 0.662 |  |
| IADL 6 month score | 0.682 | 0.658 |  |
| IADL 12 month score | 0.706 | 0.657 |  |
| Standardised Cronbach α coefficient: 0.817 | | | |
